# Exploring the potential effects of different frequencies of flashing light stimulation on alleviating mental fatigue: a behavioral and EEG study

**DOI:** 10.3389/fnhum.2026.1684714

**Published:** 2026-02-24

**Authors:** Zhenqi Liu, Aili Wei, Kang Chen, Xiujie Gao, Zilin Wei, Yingkai Qin, Bo Cui, Tianhui Wang, Kun Wang

**Affiliations:** 1 Military Medical Sciences Academy, Tianjin, China; 2 Tianjin Key Laboratory of Exercise Physiology & Sports Medicine, Tianjin University of Sport, Tianjin, China

**Keywords:** EEG, flashing light stimulation, frequency modulation, Lempel-Ziv complexity, mental fatigue

## Abstract

**Introduction:**

Mental fatigue, characterized by reduced attention and prolonged reaction times, is a critical issue in professions requiring high vigilance, such as driving and air traffic control. It contributes to numerous accidents each year. At the neural level, mental fatigue is associated with alterations in cortical oscillatory activity, particularly in the alpha and beta frequency bands, which reflect changes in alertness and cognitive engagement. While flashing light stimulation has emerged as a promising non-invasive approach to enhance alertness by modulating brain oscillations, existing research has predominantly focused on single-frequency stimulation. The potential benefits of broader frequency-band stimulation remain largely unexplored.

**Methods:**

This study aimed to explore the effects of different frequencies of flashing light stimulation on alleviating mental fatigue. A total of 32 right-handed male participants were recruited. A 2-back task was used to establish a mental fatigue model, and mental fatigue was assessed using the Psychomotor Vigilance Task (PVT) and EEG data. Participants were divided into a control group and three experimental groups, receiving flashing light stimulation at 20–30 Hz, 40 Hz, and 60 Hz for 3 minutes. EEG data were analyzed using power spectral density and Lempel-Ziv complexity (LZC) metrics to assess the effects of stimulation on brain activity.

**Results:**

The 20–30 Hz frequency-band light stimulation significantly improved reaction times and subjective fatigue ratings. EEG analysis showed normalization (decrease) of the alpha-to-beta power ratio and LZC, particularly in the frontal and occipital regions. In contrast, the 40 Hz and 60 Hz groups exhibited different EEG modulation patterns, and their effects on fatigue were more complex.

**Discussion:**

Flashing light stimulation at 20–30 Hz frequencies effectively alleviates mental fatigue and enhances alertness. These findings suggest that broader frequency-band light stimulation could offer a more effective method for reducing mental fatigue compared to single-frequency stimulation. Further research is needed to investigate the optimal parameters for light stimulation and its potential applications in clinical and everyday settings.

## 1 Introduction

Mental fatigue, characterized by a decline in attention and prolonged reaction times, arises from sustained engagement in cognitively demanding tasks (Ackerman and Kanfer, 2009; Lim and Dinges, 2008). This reduction in vigilance is especially detrimental in professions that require continuous high-level attention, such as driving, air traffic control, and medical procedures, where even brief lapses in concentration can have catastrophic consequences. In the United States alone, mental fatigue is responsible for approximately 1,000 traffic-related fatalities and over 90,000 injuries each year, highlighting its significant impact on public health (Jeon et al., 2021). Flashing light stimuli have been shown to induce the synchronization of brain oscillations associated with alertness, improve cognitive function, and influence physiological processes linked to the circadian rhythm system, enhancing overall vigilance (Huang et al., 2024; Hubbard et al., 2013; Sunde et al., 2020; Wang et al., 2024). However, despite these well-documented effects, current strategies for alleviating mental fatigue remain largely ineffective, particularly in addressing the underlying neural mechanisms. The key scientific question is whether stimulating broader frequency bands, rather than single frequencies, could provide more effective relief from mental fatigue by modulating brain oscillations more comprehensively.

Different frequencies of flashing light stimuli have varying effects on neural oscillations and cognitive function. Studies indicate that the thalamus plays a crucial role in both initial and sustained responses to light exposure during cognitive tasks, as well as in mediating the interaction between alertness and cognition (Hubbard et al., 2013; Vandewalle et al., 2009; Zhou et al., 2024). Our previous research developed a random frequency flashing light pattern between 20 and 30 Hz, which was found to enhance neural oscillations across multiple resonant frequency bands of thalamic local field potentials (LFP) (10–15 Hz, 20–30 Hz, 40–60 Hz, 60–90 Hz) (Wang et al., 2024). These brain waves, categorized by frequency and amplitude, correspond to specific cognitive and physiological states, such as alpha waves (8–12 Hz) linked to relaxed wakefulness and beta waves (12–30 Hz) associated with focused attention (Karakaş, 2020). Lower frequency flashing stimuli, within the alpha and beta ranges, have been shown to improve cognitive performance and reduce mental fatigue (Teplan et al., 2006). In coal mining operations, improving inadequate lighting can enhance cognitive work performance, influence the output efficiency of cognitive tasks, and positively affect psychological mood and alertness (Chai et al., 2024). In addition to improving cognitive function, flashing light stimuli have demonstrated health benefits, such as 40 Hz light stimulation, which induces gamma oscillations. These oscillations have been shown to promote microglial aggregation and reduce amyloid plaques in the medial prefrontal cortex (Martorell et al., 2019). Furthermore, 40 Hz stimulation can increase aquaporin-4 polarization in astrocytes and enhance vasomotion, improving lymphatic inflow and outflow, with potential implications for treating neurological diseases (Sun et al., 2024). While single-frequency stimulation has yielded valuable insights, the purpose of this study is to explore the effects of broader frequency bands on mental fatigue. Multi-frequency stimulation could more effectively target a range of neural oscillations, potentially providing a more robust solution for cognitive recovery and fatigue reduction (van Driel et al., 2015). Thus, further research is necessary to investigate how broader frequency band stimulation can influence mental fatigue.

The significance of this study lies in its potential to reveal new strategies for mitigating mental fatigue by utilizing broader frequency bands of flashing light stimulation. This approach could provide a novel theoretical foundation for developing more effective interventions to enhance cognitive performance and reduce mental fatigue, with practical applications in both clinical and professional settings.

## 2 Materials and methods

### 2.1 Participants

Participants comprised 32 right-handed males, aged between 18 and 26 years, all with normal or corrected-to-normal vision and without any color vision deficiencies. Only male participants were enrolled to minimize potential confounding from sex-related variability in fatigue perception and cortical recruitment patterns, as sex differences have been reported in both susceptibility to mental fatigue and the propensity to report fatigue-related subjective symptoms (Wylie et al., 2022; Vymyslický et al., 2022). Prior to inclusion, all participants underwent a comprehensive health screening to rule out any cardiac, pulmonary, or neurological disorders. Strict pre-experimental conditions were enforced: participants abstained from stimulants including alcohol and any medications for a week leading up to the experiment, maintained a consistent sleep schedule, and avoided strenuous physical or cognitive exertions. On the experimental day, psychological assessments confirmed all participants were in an optimal mental state, ensuring standardized baseline conditions for cognitive performance measurements.

### 2.2 Experimental procedure

**Figure 1:**
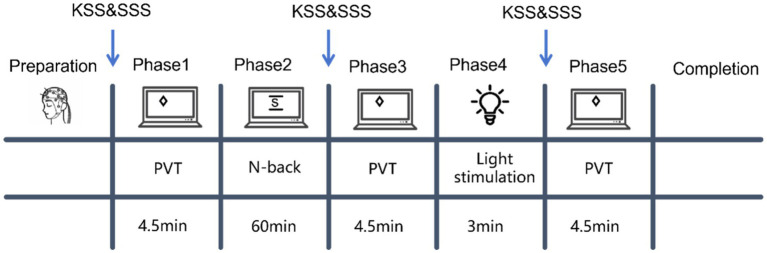
Schematic overview of the experimental procedure. The experiment consisted of five phases: Phase 1 (baseline PVT, 4.5 min), Phase 2 (N-back task for fatigue induction, 60 min), Phase 3 (post-fatigue PVT, 4.5 min), Phase 4 (light stimulation or rest for Control group, 3 min), and Phase 5 (post-stimulation PVT, 4.5 min). Subjective sleepiness was assessed using the Karolinska Sleepiness Scale (KSS) and Stanford Sleepiness Scale (SSS) at phases 1, 3, and 5 (indicated by arrows). PVT, psychomotor vigilance task.

The sample size of 32 participants (8 per group across four conditions: Control, 20–30 Hz, 40 Hz, and 60 Hz) was determined based on previous EEG studies investigating the effects of visual stimulation on mental fatigue and cognitive performance. Prior studies employing comparable experimental paradigms—including flashing light stimulation with EEG recordings, sustained cognitive tasks for fatigue induction, and within-subject repeated-measures designs—have typically recruited 8–31 participants and reported significant within-subject effects on EEG spectral metrics (alpha, beta, theta power) and behavioral vigilance measures (Li et al., 2020; Hamann and Carstengerdes, 2023; Cao et al., 2014). Specifically, Li’s study employed 20 male participants for EEG-based mental fatigue assessment using sustained cognitive tasks (Li et al., 2020); Hamann’s study recruited 31 participants to evaluate mental fatigue development using concurrent EEG-fNIRS during a 90-min simulated flight task (Hamann and Carstengerdes, 2023); and studies on SSVEP-based fatigue detection have successfully demonstrated significant EEG changes with sample sizes of 8–13 participants per condition (Cao et al., 2014). Given our repeated-measures design with multiple within-session assessment phases (Phases 1, 3, and 5) and the use of non-parametric statistical tests (Wilcoxon signed-rank test) for paired within-subject comparisons, this sample size was considered adequate to detect medium-to-large within-group effects while maintaining practical feasibility (Figure 1).

### 2.3 Experimental paradigm

#### 2.3.1 N-back task (2-back version)

The N-back task, designed to assess working memory, was conducted using PsychoPy (version 2024.1.4) on a system running Windows 10. Visual stimuli were presented on a 27-inch Dell D2721H monitor (Dell Inc., USA; 1,920 × 1,080 resolution, 60 Hz refresh rate, IPS panel). Participants were seated at an approximate viewing distance of 60 cm from the screen in a controlled, dimly-lit environment (Owen et al., 2005).

Each trial displayed a single uppercase English letter randomly chosen from the alphabet, presented in Arial font size 40 within a centrally-located gray box on the screen. Participants were required to respond by pressing the ‘S’ key if the displayed letter matched the one shown two trials previously, or the ‘L’ key if it did not match. The response window was limited to 1,500 ms post-stimulus presentation.

Feedback was immediate: a green border appeared around the box for 500 ms following a correct response, while a red border signaled an incorrect or missed response. The inter-stimulus interval varied randomly between 2.0 and 2.5 s to mitigate anticipatory responses.

The fatigue-induction task consisted of a 60-min N-back task (2-back version) administered in multiple consecutive blocks to maintain sustained cognitive load while allowing brief inter-block transitions (Borragán et al., 2017). Trials were presented in a randomized order with an approximately balanced proportion of match and non-match events to minimize sequence effects. The task software recorded trial-by-trial reaction time and accuracy, which were used for subsequent analyses of working-memory performance and task-induced fatigue.

#### 2.3.2 PVT experimental paradigm

The Psychomotor Vigilance Task (PVT) quantified participants’ alertness and reaction speed over a 4.5-min period. During the task, a yellow diamond was displayed for 50 ms at random intervals ranging from 1.5 to 4 s on a computer screen (Dinges and Powell, 1985). Participants responded by pressing the space bar promptly upon stimulus presentation. The task included 100 trials, with responses exceeding 500 ms or prior to stimulus onset categorized as errors. This design rigorously measures the sustained attention capacity of participants under controlled experimental conditions.

#### 2.3.3 Stimulus setting

This stimulation was administered using a precision-controlled LED white light source, specifically the SENNO-HSL-39536 model (Shenzhen Hexingwei Automation Technology, Shenzhen, China). The frequency of the light flashes was meticulously regulated by a microcontroller (DFRduino UNO R3 development board) and a constant current light source controller (BS-DC120-IO3CE-24010-4, Shenzhen Hexingwei Automation Technology), ensuring consistent delivery across all experimental sessions.

### 2.4 Data processing

EEG data were acquired using the Okti128 system (Compumedics, Australia), equipped with 128 electrodes placed according to the international 10–20 system, covering major scalp regions including frontal, central, temporal, parietal, and occipital areas. The system recorded data with a sampling rate of 1,000 Hz and a 24-bit depth. EEG recording was synchronized with the PsychoPy task using the system’s accompanying SyncBox, which transmitted TTL trigger signals at stimulus onset and response events to ensure precise temporal alignment between behavioral events and neural recordings.

EEG data preprocessing was critical for ensuring high-quality analyses. The processing pipeline, implemented in MATLAB using the EEGLAB toolbox (Delorme and Makeig, 2004), involved several steps: First, signals contaminated with electrooculographic (EOG) artifacts were removed. The data were then re-referenced to the average of bilateral mastoid signals to standardize the electrical reference point across all recordings. A band-pass filter was applied with cutoffs set from 0.1 to 50 Hz, and the sampling frequency was reduced from 1,000 Hz to 500 Hz to decrease data redundancy without losing essential information. Data segments corresponding to experimental Phases 1, 3, 4, and 5 were isolated, and Independent Component Analysis (ICA) was performed using the Infomax algorithm to identify and remove non-brain artifacts; artifactual independent components (ICs) were identified through visual inspection of topographic maps, time courses, and power spectra, with an average of 8.2 ± 2.4 ICs (range: 4–14) rejected per participant. Power spectral densities were estimated using the Welch method, and Lempel-Ziv Complexity (LZC) was calculated to measure the EEG signal complexity, providing insights into neural dynamics during different experimental conditions.

#### 2.4.1 Pwelch

Power spectral density (PSD) was estimated using the Welch method, which has been widely applied in EEG-based mental fatigue research to quantify frequency-domain characteristics of neural oscillations (Gharagozlou et al., 2015). For each electrode and phase, PSD was computed from artifact-free, continuous EEG segments using the MATLAB pwelch function with the following parameters: a 2-s Hamming window (1,000 samples), 50% overlap between adjacent windows, and 2048-point FFT resolution. Alpha power (Eα) was defined as the integrated PSD in the 8–13 Hz range, and beta power (Eβ) was defined as the integrated PSD in the 13–30 Hz range. Band power was normalized to total power (1–50 Hz) to obtain relative power values, reducing inter-individual variability in absolute power. The alpha-to-beta power ratio (Eα/Eβ) was then calculated for each electrode. For each participant, electrode-level PSD values were computed for each experimental phase and used directly in subsequent statistical analyses; region-wise averages were additionally computed by averaging across electrodes within predefined scalp regions (frontal, central, temporal, parietal, and occipital) for visualization purposes.

#### 2.4.2 LZC

LZC quantifies the algorithmic complexity of an EEG time series by counting the number of distinct subsequences that appear as the sequence is parsed from left to right, with larger values indicating less repetitive and more information-rich temporal patterns (Vymyslický et al., 2022). For EEG applications, LZC is typically computed using an efficient parsing procedure and is normalized to reduce sequence-length dependence (Wylie et al., 2022). LZC has been widely used to index state-dependent changes in EEG signal diversity across variations in vigilance and cognitive demand (Miley et al., 2016; MacLean et al., 1992; Zhang et al., 2021). While several established alternatives exist—including entropy-based indices such as approximate entropy (ApEn) (Pincus et al., 1991) and sample entropy (SampEn) (Richman and Moorman, 2000), multiscale extensions such as multiscale entropy (MSE) (Costa et al., 2002) and composite multiscale entropy (CMSE) (Wu et al., 2013), and more recent dynamic entropy frameworks (Zavala-Yoe and Ramirez-Mendoza, 2017; Zavala-Yoé et al., 2023)—we selected normalized LZC as a parsimonious primary index because it is parameter-light and reproducible across channels and participants, can be estimated efficiently in a window-based manner from fixed-length artifact-free segments, and avoids additional scale- or model-selection steps and longer effective data-length requirements that are often needed for multiscale or dynamic approaches. Accordingly, LZC was used here to complement spectral metrics as a reproducible summary of EEG signal diversity under a repeated-measures design.

For each electrode and phase, LZC was computed from artifact-free, continuous EEG data after preprocessing. Signals were downsampled to 500 Hz, band-pass filtered at 0.1–50 Hz with no notch filtering, and re-referenced to the mastoids (see Section 2.4 for artifact correction procedures). For each phase, an artifact-free segment of 120 s was extracted from the central portion of the phase, excluding the initial and terminal transition periods to reduce non-stationarity related to phase onset/offset (Zhang et al., 2021). The selected segment was z-scored within each channel. The signal was binarized using the segment median as the threshold: samples above the median were coded as “1” and samples at or below the median were coded as “0,” yielding a binary sequence *S* of length *n* (Wylie et al., 2022; Miley et al., 2016).

The binarized sequence was divided into non-overlapping windows of 2,000 samples (4 s), and LZC was computed for each window using the Kaspar–Schuster implementation of the Lempel–Ziv parsing algorithm (Wylie et al., 2022; Vymyslický et al., 2022). Normalized LZC was calculated as:


$$\mathrm{LZC}=\frac{c}{n/\log_{2}(n)}$$

where *c*(*n*) is the number of distinct subsequences identified by the parsing procedure and *n* is the length of the binary sequence (Vymyslický et al., 2022; Cao et al., 2014). Window-level LZC values were averaged to obtain one LZC estimate per electrode per phase, which was used for subsequent statistical analyses.

### 2.5 Statistical evaluation of mental fatigue metrics

We evaluated changes in mental fatigue across Phases 1, 3, and 5 using subjective ratings from the KSS and SSS and objective performance indices from the psychomotor vigilance task (PVT; reaction time) (Åkerstedt and Gillberg, 1990; Kaida et al., 2006; Hoddes et al., 1973). For analyses of subjective outcomes, a combined subjective sleepiness score (KSS + SSS) was additionally computed. EEG-derived metrics, including the alpha-to-beta power ratio (Eα/Eβ) and LZC, were analyzed to characterize phase-related changes in neural activity.

Phase-related changes within each condition were assessed using the Wilcoxon signed-rank test for paired within-subject comparisons. For electrode-wise EEG analyses, *p* values were corrected for multiple comparisons across electrodes using the Benjamini–Hochberg false discovery rate (FDR) procedure, and statistical significance was evaluated using FDR-adjusted *p* values (*q* values) with *q* < 0.05.

To test ordered frequency-related trends in behavioral and subjective fatigue outcomes, we applied the Jonckheere–Terpstra trend test to phase-change scores (Phase5–Phase3) for PVT reaction time and the combined subjective sleepiness score (KSS + SSS) across stimulation-frequency conditions (Jonckheere, 1954).

To further characterize EEG frequency dynamics during stimulation, alpha (Eα) and beta (Eβ) band power were computed at 1-s resolution within 120-s segments from the stimulation and rest periods. For each participant and condition, band power was first averaged across all electrodes at each time point, yielding a single time series per frequency band. Ordinary least squares (OLS) linear regression models were then fitted to these second-by-second band-power time series, with time (in seconds) as the independent variable, to quantify time-dependent changes in spectral power during stimulation. Goodness-of-fit was assessed using *R*^2^, and statistical significance of the slope was evaluated using *F*-tests. This within-stimulation temporal analysis was included to provide a complementary, process-level perspective on how neural oscillations evolved during the stimulation interval, as prior studies have similarly employed time-resolved spectral analyses to characterize dynamic changes in alpha and beta power during vigilance tasks (Harvy et al., 2022; Engelbregt et al., 2021). It should be noted that standard OLS regression assumes independence of residuals, which may be violated in time-series data due to autocorrelation; this limitation is acknowledged in the Discussion.

Fleiss’ kappa agreement analysis. To provide a complementary classification-based perspective on the consistency of recovery effects across multiple fatigue-related measures, we conducted an exploratory Fleiss’ kappa (*κ*) agreement analysis on discretized phase-change outcomes. For each participant, recovery/change scores were computed as Δ = Phase5 − Phase3 for PVT reaction time, the combined subjective sleepiness score (KSS + SSS), Eα/Eβ, and LZC. For EEG-derived measures (Eα/Eβ and LZC), participant-level values were obtained by averaging across all electrodes within each phase prior to computing Δ. Each Δ score was then dichotomized into Improved versus Not improved using a pre-specified zero-threshold rule: for PVT reaction time, Improved was defined as ΔRT < 0 (faster responses); for KSS + SSS, Improved was defined as Δ(KSS + SSS) < 0 (reduced sleepiness); for Eα/Eβ, Improved was defined as Δ(Eα/Eβ) < 0; and for LZC, Improved was defined as ΔLZC > 0. Within each stimulation-frequency group, these four metrics were treated as independent “raters” assigning categorical labels to each participant, and Fleiss’ *κ* was computed to quantify inter-metric agreement in the direction of recovery. This agreement analysis was included as an additional complementary approach to summarize the consistency of recovery directions across multiple metrics; inferential testing of group effects remained based on the prespecified Wilcoxon/Jonckheere–Terpstra analyses.

## 3 Results

### 3.1 20–30 Hz flashing light stimulation improves reaction time and reduces subjective fatigue

**Figure 2:**
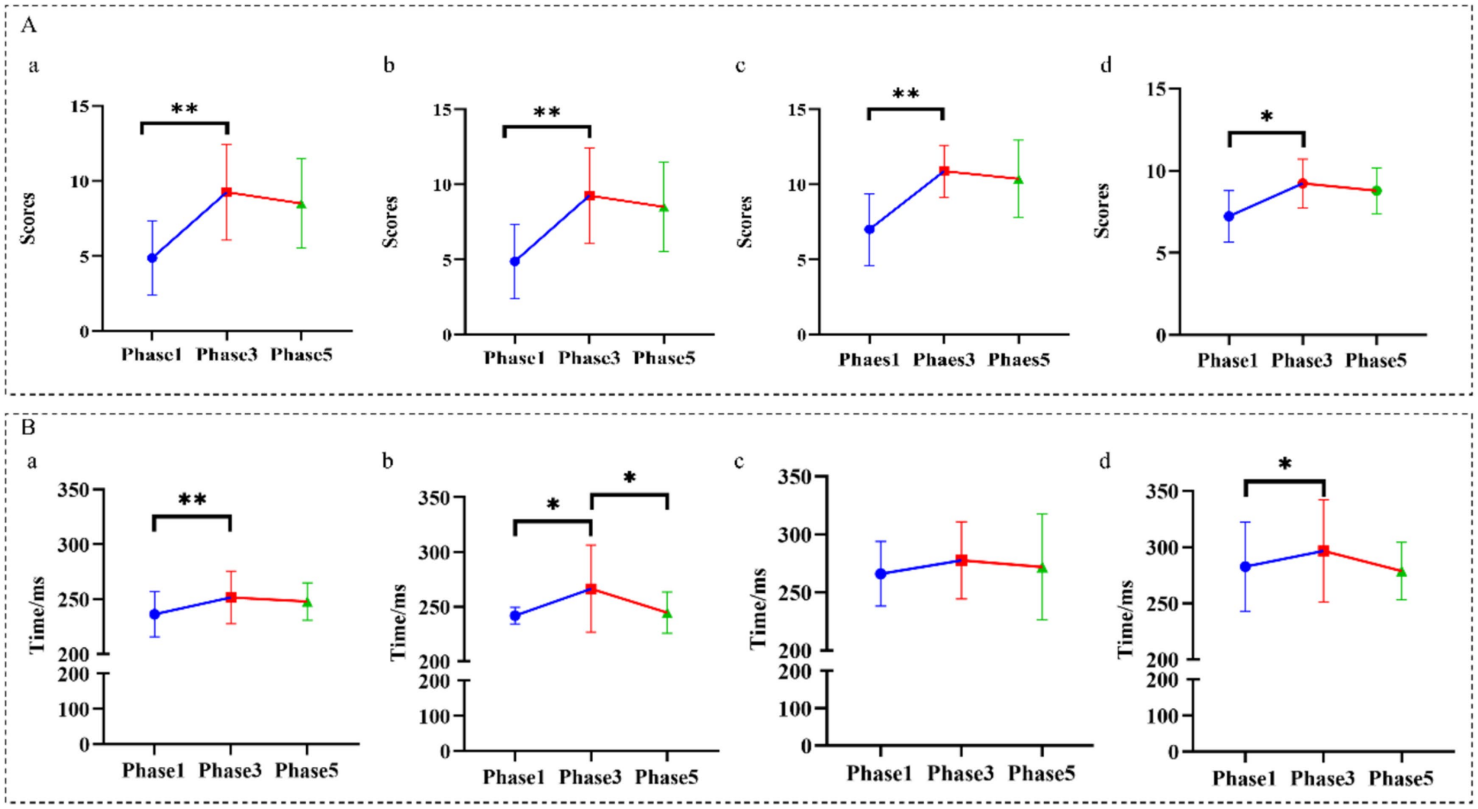
Behavioral experiment results. **(A)** Combined subjective sleepiness score (KSS + SSS) for the control **(a)**, 20–30 Hz **(b)**, 40 Hz **(c)**, and 60 Hz **(d)** groups measured at three time points: pre–N-back (Phase 1), post–N-back/pre-stimulation (Phase 3), and post-stimulation (Phase 5). **(B)** Psychomotor vigilance task (PVT) reaction time measured at the same time points for each group. Statistical methods are described in Section 2.5. Data are presented as mean ± SEM. *p < 0.05, **p < 0.01.

Across all groups, KSS and SSS scores increased from Phase 1 to Phase 3, indicating successful fatigue induction by the 1-h N-back task (Figure 2A). From Phase 3 to Phase 5, subjective scores showed a downward trend; however, these post-stimulation changes did not reach statistical significance in within-group paired comparisons (Figure 2A). In parallel, PVT reaction time increased from Phase 1 to Phase 3, with significant within-group increases observed in the Control, 20–30 Hz, and 60 Hz groups (Figure 2B). Notably, following stimulation (Phase 3 to Phase 5), the 20–30 Hz group exhibited a significant reduction in PVT reaction time, whereas no statistically significant post-stimulation change was detected in the other groups (Figure 2B).

### 3.2 20–30 Hz stimulation normalizes Eα/Eβ and modulates LZC

**Figure 3:**
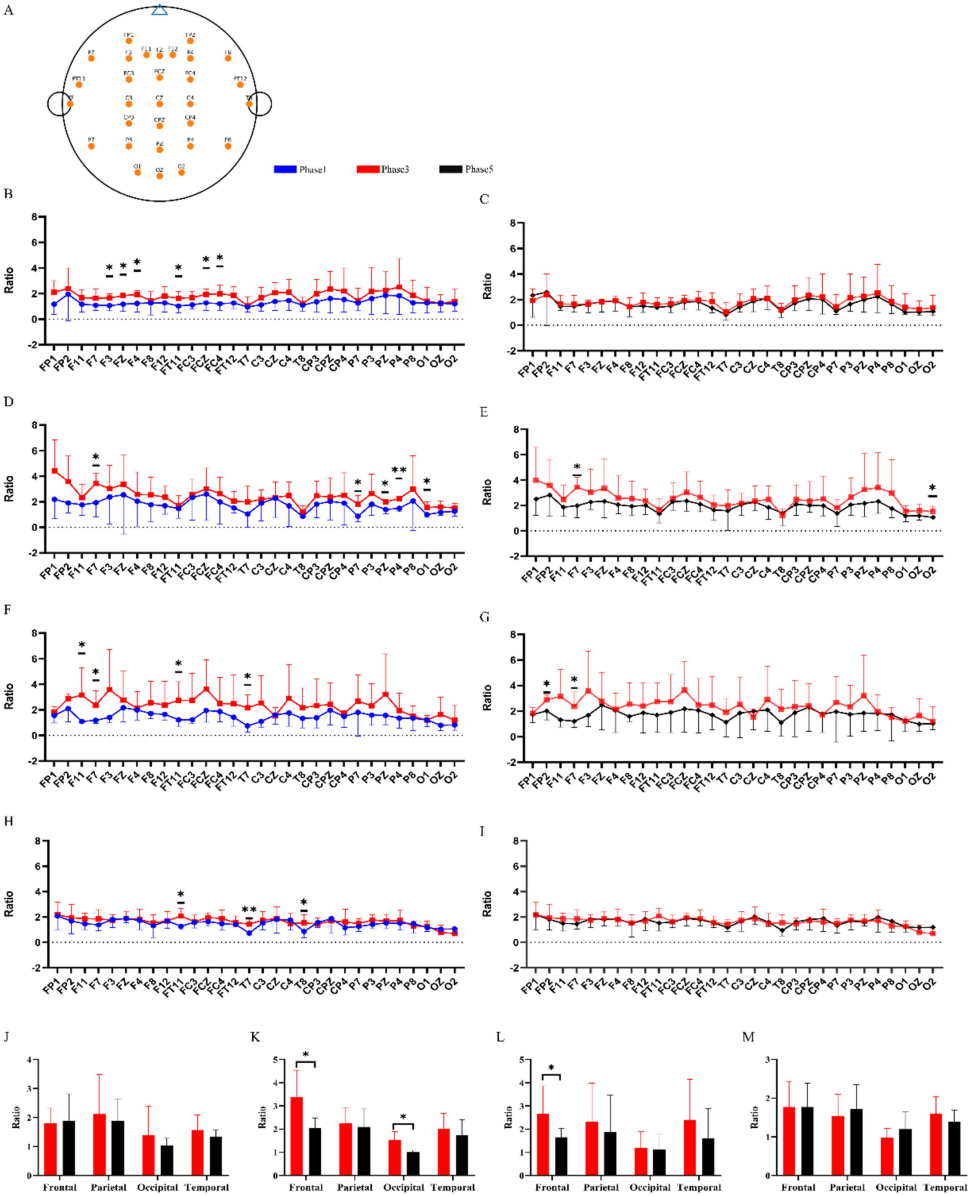
Spatially referenced and region-wise changes in the EEG alpha-to-beta power ratio (Eα/Eβ) across experimental phases and stimulation conditions. **(A)** Schematic layout of the analyzed EEG electrodes based on the international 10–20 system; orange markers denote channels included in the statistical analysis. **(B–I)** Electrode-wise Eα/Eβ for the Control **(B,C)**, 20–30 Hz **(D,E)**, 40 Hz **(F,G)**, and 60 Hz **(H,I)** groups. For each condition, the left panel compares Phase 1 (pre–N-back) versus Phase 3 (post–N-back/pre-stimulation), and the right panel compares Phase 3 versus Phase 5 (post-stimulation). Electrode labels are shown on the *x*-axis. **(J–M)** Region-wise Eα/Eβ averaged within four predefined scalp regions (frontal, parietal, occipital, and temporal) for the Control **(J)**, 20–30 Hz **(K)**, 40 Hz **(L)**, and 60 Hz **(M)** groups, shown for Phase 3 and Phase 5. Statistical methods and multiple comparison corrections are described in Section 2.5. Data are presented as mean ± SEM. *q < 0.05, **q < 0.01.

**Figure 4:**
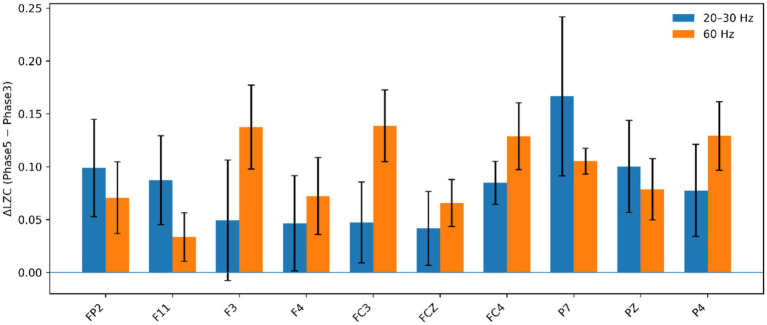
Electrode-level comparison of recovery-related changes in LZC between stimulation conditions. LZC was computed for each electrode at Phase 3 (post–N-back/pre-stimulation) and Phase 5 (post-stimulation). Recovery-related changes were quantified as ΔLZC = Phase5 − Phase3. Bars show mean ΔLZC (± SEM) for the 20–30 Hz and 60 Hz stimulation conditions at each electrode. Statistical methods are described in Section 2.5. No electrodes showed significant between-condition differences after FDR correction (all *q* ≥ 0.05).

As shown in Figure 3, Eα/Eβ increased from Phase 1 to Phase 3 across multiple electrodes in all conditions. During the recovery interval (Phase 3 to Phase 5), the 20–30 Hz condition exhibited significant normalization of Eα/Eβ at 2 of 30 electrodes (F7 and O2; *q* < 0.05, FDR-corrected). Region-level analyses revealed significant Phase 3-to-Phase 5 decreases in Eα/Eβ in the frontal ROI (3.37 ± 0.48 to 2.04 ± 0.18; *p* = 0.026) and occipital ROI (1.54 ± 0.16 to 1.01 ± 0.04; *p* = 0.013) (Figures 3D,E,K). The Control condition showed no significant Phase 3-to-Phase 5 changes (Figures 3B,C,J), and the 40 Hz and 60 Hz conditions similarly did not show significant Phase 3-to-Phase 5 changes at either electrode or ROI levels (Figures 3F–I,L, M). Given that Phase 3-to-Phase 5 changes in LZC were evident only in the 20–30 Hz and 60 Hz conditions, subsequent electrode-level analyses focused on recovery-related complexity modulation quantified as ΔLZC = Phase5 − Phase3 (Figure 4); although ΔLZC was positive at several electrodes in both conditions, no between-condition differences survived FDR correction (all *q* ≥ 0.05).

### 3.3 The increase of *α* and the decrease of *β* during stimulation

**Figure 5:**
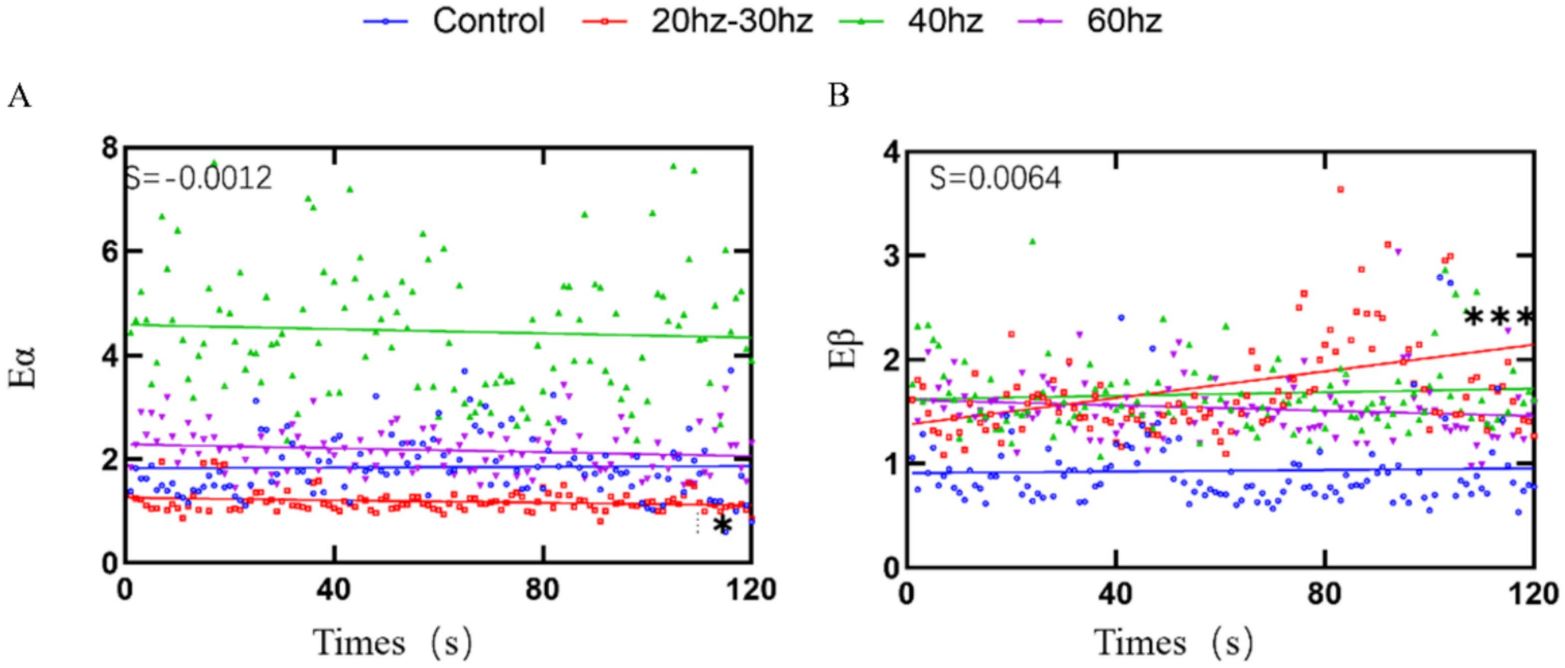
Time-resolved linear regression of EEG alpha and beta power during stimulation. **(A)** Eα over the first 120 s of stimulation is shown for each condition, with overlaid linear regression fits to illustrate temporal trends. **(B)** Eβ over the first 120 s of stimulation with corresponding linear regression fits. S indicates the estimated slope for the 20–30 Hz condition. Statistical methods are described in Section 2.5. *p < 0.05, **p < 0.01, ***p < 0.001.

Linear regression was performed on Eα and Eβ band power over the first 120 s of stimulation for each condition (Figure 5). Regression parameters are summarized in Table 1.

**Table 1:** Linear regression parameters for within-stimulation EEG power dynamics.

Band	Condition	Intercept	Slope (S)	*R* ^2^	*F*(1,118)	*p*
Alpha (Eα)	Control	1.827	0.00035	<0.001	0.052	0.819
	20–30 Hz	1.259	−0.00119	0.040	4.893	0.029*
	40 Hz	4.587	−0.00206	0.004	0.419	0.519
	60 Hz	2.287	−0.00188	0.023	2.772	0.099
Beta (Eβ)	Control	0.910	0.00037	0.001	0.138	0.711
	20–30 Hz	1.376	0.00641	0.113	15.03	0.0002**
	40 Hz	1.619	0.00086	0.005	0.617	0.434
	60 Hz	1.614	−0.00129	0.026	3.106	0.081

OLS linear regression was performed on second-by-second band power (averaged across electrodes) over the first 120 s of stimulation. Time (in seconds) served as the independent variable. Intercept and slope values are expressed in relative power units. *p < 0.05, **p < 0.001.

In the alpha band, only the 20–30 Hz condition showed a statistically significant negative temporal trend (slope *S* = −0.00119, *R*^2^ = 0.040, *F*(1,118) = 4.89, *p* = 0.029). The other conditions displayed non-significant alpha trends: Control (*S* = 0.00035, *R*^2^ < 0.001, *p* = 0.819), 40 Hz (*S* = −0.00206, *R*^2^ = 0.004, *p* = 0.519), and 60 Hz (*S* = −0.00188, *R*^2^ = 0.023, *p* = 0.099) (Figure 5A).

In contrast, beta power showed a significant positive temporal trend only in the 20–30 Hz condition (slope *S* = 0.00641, *R*^2^ = 0.113, *F*(1,118) = 15.03, *p* = 0.0002). The other conditions exhibited non-significant beta trends: Control (*S* = 0.00037, *R*^2^ = 0.001, *p* = 0.711), 40 Hz (*S* = 0.00086, *R*^2^ = 0.005, *p* = 0.434), and 60 Hz (*S* = −0.00129, *R*^2^ = 0.026, *p* = 0.081) (Figure 5B).

### 3.4 Cross-metric agreement analysis (Fleiss’ *κ*)

As summarized in Table 2, Fleiss’ *κ* values were low across conditions (*κ* values near 0 with 95% confidence intervals spanning 0), indicating limited cross-metric concordance in recovery direction at the participant level. Although the overall proportion of “Improved” labels was relatively high in some conditions, the expected agreement by chance was also high, resulting in *κ* estimates close to zero.

**Table 2:** Cross-metric agreement in recovery direction across fatigue-related outcomes assessed by Fleiss’ *κ*.

Condition	*n*	Fleiss’ *κ*	95% CI (low, high)	Observed agreement P̄	Expected agreement Pe	Overall improved proportion	PVT improved *n* (%)	Subjective improved *n* (%)	Eα/Eβ improved *n* (%)	LZC improved *n* (%)
Control	8	0.04895	−0.25714, 0.33333	0.52778	0.50347	0.45833	37.50	75.00	37.50	50.00
20–30 Hz	8	−0.00840	−0.33333, 0.25000	0.58333	0.58681	0.70833	87.50	50.00	75.50	87.50
40 Hz	8	0.12500	−0.24444, 0.41053	0.61111	0.55556	0.66667	87.50	50.00	87.50	50.00
60 Hz	8	~0.00000	−0.25000, 0.27273	0.72222	0.72222	0.83333	100.00	75.00	75.00	100.00

Δ denotes Phase5–Phase3 change. “Improved” was defined as ΔRT < 0 for PVT reaction time, Δ(Subjective) < 0 for the combined subjective sleepiness score (KSS+SSS), Δ(Eα/Eβ) < 0 for the alpha-to-beta power ratio, and ΔLZC > 0 for normalized Lempel–Ziv complexity (LZC). Fleiss’ *κ* quantifies inter-metric agreement in the direction of recovery within each condition. P̄ indicates the observed agreement and Pe indicates the agreement expected by chance given the marginal category proportions. Overall improved proportion represents the fraction of “Improved” labels across all participants and all four metrics within each condition. 95% confidence intervals for κ were obtained by bootstrap resampling.

## 4 Discussion

Using a controlled fatigue-induction and recovery paradigm, we evaluated the effects of frequency-specific flashing light stimulation on mental fatigue by integrating subjective ratings (KSS and SSS), objective vigilance performance (PVT reaction time), and EEG-derived indices reflecting spectral balance and signal diversity (Eα/Eβ and LZC). Across all conditions, the 1-h N-back task reliably increased fatigue, as indicated by elevated subjective sleepiness and slower vigilance responses from Phase 1 to Phase 3, supporting successful induction of mental fatigue by sustained cognitive load. During the recovery interval (Phase 3 to Phase 5), the behavioral data suggested that recovery was not uniform across frequencies: while subjective ratings tended to decrease after the stimulation/rest period, these changes were modest and did not consistently reach within-condition significance, whereas objective vigilance showed clearer modulation, with the 20–30 Hz condition demonstrating the most evident post-stimulation improvement in PVT reaction time. This dissociation between subjective and objective outcomes is consistent with the notion that self-reported sleepiness is sensitive to expectancy, interoceptive awareness, and response tendencies, and may show limited resolution for detecting subtle short-term recovery effects compared with performance-based metrics (Miley et al., 2016; MacLean et al., 1992; Zhang et al., 2021; Myllylä et al., 2022; Mankowska et al., 2021; Liu et al., 2022). Consistent with this multidimensional profile, a cross-metric agreement analysis based on discretized Phase5–Phase3 changes indicated low concordance across subjective, behavioral, and EEG-derived endpoints (Table 2), suggesting that these measures capture partially distinct facets of fatigue recovery rather than a single unified “improvement” response.

EEG analyses provided convergent physiological context for these behavioral observations. Prior work supports EEG power as a sensitive index of fatigue-related state changes and suggests that ratio-based metrics can outperform single-band measures by capturing the balance between slower and faster oscillatory activity (Harvy et al., 2022; Huang et al., 2016; Jap et al., 2009; Chen et al., 2013; Xu et al., 2018). In the present dataset, the alpha-to-beta power ratio (Eα/Eβ) increased from Phase 1 to Phase 3 across many electrodes in all groups, consistent with fatigue-associated spectral shifts following sustained cognitive engagement. Importantly, after the recovery interval (Phase 3 to Phase 5), the 20–30 Hz condition exhibited a more apparent normalization of Eα/Eβ relative to other conditions, with region-level differences most evident in frontal and occipital regions, whereas the Control condition showed comparatively limited change and the 40 Hz and 60 Hz conditions displayed smaller and less consistent Phase 3–to–Phase 5 modulation. The regional specificity of Eα/Eβ normalization—particularly in frontal and occipital areas—is consistent with the known neuroanatomical substrates of fatigue and visual processing. Frontal regions, especially the prefrontal cortex, are critically involved in executive control, sustained attention, and the subjective experience of mental fatigue, and have been repeatedly implicated in fatigue-related EEG changes during prolonged cognitive tasks (Boksem et al., 2005; Lim et al., 2010). Occipital regions, as the primary visual cortex, are the initial cortical target of photic stimulation and would be expected to show robust entrainment-related modulation during flashing light exposure (Herrmann, 2001). The convergence of recovery-related Eα/Eβ changes in both frontal and occipital ROIs may therefore reflect a pathway in which visual stimulation at 20–30 Hz engages occipital circuits through photic driving, with downstream modulation of frontal networks involved in vigilance regulation. However, it should be noted that electrode-level analyses revealed considerable inter-individual and inter-electrode variability, particularly for LZC, where recovery-related changes were heterogeneous across scalp locations and did not survive multiple-comparison correction. This spatial heterogeneity may reflect individual differences in cortical anatomy, fatigue susceptibility, or the extent of stimulation-induced entrainment, and underscores the need for larger samples and potentially source-level analyses to delineate the precise neural circuits mediating frequency-specific effects. Although the specific neurophysiological interpretation of Eα/Eβ changes can vary with task context, these results collectively support a frequency-dependent pattern in which 20–30 Hz stimulation is associated with a more prominent shift toward a less fatigue-like spectral balance during recovery, aligning with the objective behavioral benefit observed in PVT performance. For the higher-frequency conditions, the absence of clear post-stimulation normalization could reflect weaker entrainment efficacy, greater inter-individual variability, or perceptual factors that alter the effective stimulation dose; for example, flicker fusion at higher frequencies may reduce the salience of the visual stimulus and thereby attenuate physiological impact, although this interpretation should be considered tentative without direct perceptual quantification (Mankowska et al., 2021).

We also assessed neural signal complexity using LZC, which has been proposed as an EEG feature sensitive to state-dependent changes in information diversity and has been applied in fatigue-related contexts (Diez et al., 2024; Ibáñez-Molina et al., 2015; Li et al., 2008; Liu et al., 2010). In our phase-level analyses, LZC differed between Phase 1 and Phase 3 at selected electrodes, indicating that sustained cognitive load was accompanied by measurable changes in signal diversity. However, when recovery-related complexity modulation was quantified as ΔLZC (Phase5 − Phase3) and evaluated at the electrode level, the between-frequency differences were not robust after correction for multiple comparisons, despite positive ΔLZC at several electrodes in both the 20–30 Hz and 60 Hz conditions. This pattern suggests that complexity changes during recovery were heterogeneous across scalp locations and participants and that any frequency-specific effect on LZC, if present, was subtle under the current sample size and analytic granularity. Consequently, LZC in this study should be interpreted as a complementary and exploratory index rather than definitive evidence for frequency specificity, whereas the convergent changes in objective vigilance and Eα/Eβ provide the primary support for the 20–30 Hz benefit. Notably, consistent with this interpretation, Table 1 shows that cross-metric agreement in the direction of recovery was low (Fleiss’ *κ* values near 0 with 95% confidence intervals spanning 0), underscoring that LZC- and Eα/Eβ-derived categorizations do not uniformly match behavioral and subjective categorizations at the individual level.

To further link post-stimulation outcomes to stimulation-period physiology, we analyzed time-resolved EEG power dynamics during the first 120 s of stimulation. This within-stimulation analysis showed that the 20–30 Hz condition exhibited the clearest monotonic decrease in alpha power and increase in beta power over time, whereas the other conditions showed comparatively modest or less consistent trends. Such an α-down/β-up pattern is directionally consistent with increased cortical activation and vigilance (Engelbregt et al., 2021) and provides a process-level complement to the phase-based recovery effects observed in behavioral performance and Eα/Eβ. Notably, this analysis describes temporal modulation during stimulation rather than a pre/post contrast, and therefore it should be interpreted as evidence that the 20–30 Hz condition is associated with a more pronounced real-time oscillatory shift during the stimulation interval, which may contribute to the subsequent recovery profile.

Several limitations should be considered. First, the male-only sample improves internal control but limits generalizability to females, and future studies should explicitly evaluate sex as a biological variable in both subjective and EEG outcomes (Wylie et al., 2022; Vymyslický et al., 2022). Second, although the sample size (n = 8 per group) was determined based on comparable EEG studies investigating visual stimulation and mental fatigue effects (Li et al., 2020; Hamann and Carstengerdes, 2023; Cao et al., 2014), it nonetheless constrains statistical power, particularly for electrode-wise analyses that require multiple-comparison correction; this limitation is most relevant for complexity metrics such as LZC, which can be sensitive to inter-individual variability and methodological choices (Diez et al., 2024; Ibáñez-Molina et al., 2015; Li et al., 2008; Liu et al., 2010). Larger samples would improve the precision of effect estimates and enable more robust between-group comparisons. Third, the cross-metric agreement analysis required dichotomization of continuous outcomes (Improved vs. Not improved), which may reduce sensitivity and contribute to wider uncertainty in *κ* estimates, especially with modest group sizes (Table 2). Fourth, while the stimulation-frequency manipulation was controlled, perceptual factors such as flicker fusion and subjective comfort were not quantified, which restricts mechanistic inference regarding effective stimulation dose at higher frequencies (Mankowska et al., 2021). Fifth, the current study focused on short-term recovery within a single session; longer follow-up windows and repeated-session designs will be important to determine durability and potential habituation effects. Sixth, the within-stimulation linear regression analysis employed OLS regression on time-series data, which assumes independence of residuals. This assumption may be violated due to temporal autocorrelation in successive power estimates. Although the primary conclusions regarding the 20–30 Hz condition are supported by statistically significant effects (Table 1), future studies could employ time-series regression approaches (e.g., autoregressive models or Newey-West heteroskedasticity and autocorrelation consistent standard errors) to more rigorously account for temporal dependencies. Finally, although EEG provides temporally precise signatures, source-level analyses or multimodal measurements may be required to delineate the neural circuits mediating frequency-specific effects.

Future work should therefore (i) increase sample size and include both sexes; (ii) preregister primary endpoints and analytic plans, particularly for multichannel EEG complexity measures; (iii) incorporate perceptual and physiological covariates that index effective stimulation (e.g., subjective flicker perception, discomfort, pupil dynamics); (iv) test a broader frequency range and individualized parameter tuning to optimize efficacy; and (v) extend the design to repeated sessions and ecologically relevant fatigue settings. Taken together, the present findings indicate that flashing light stimulation can modulate fatigue-related behavioral and electrophysiological signatures, with 20–30 Hz showing the most consistent convergence between objective vigilance improvement and EEG spectral normalization, supporting its potential as a noninvasive approach for facilitating short-term recovery from mental fatigue. In parallel, the low cross-metric agreement suggests that future studies should define primary endpoints *a priori* and consider multivariate or composite approaches to capture recovery more holistically.

## 5 Conclusion

The findings of this study indicate that frequency-specific flashing light stimulation can modulate fatigue-related electrophysiological signatures. Among the tested conditions, 20–30 Hz stimulation showed the most consistent evidence for short-term recovery, with improved objective vigilance and normalization of Eα/Eβ. Cross-metric agreement across outcomes was limited (Table 2), highlighting the complementary nature of behavioral, subjective, and EEG-derived measures. Future studies should validate these effects in larger and more diverse samples, refine stimulation parameters, and clarify underlying mechanisms to support translation to real-world and clinical contexts.

## Data Availability

The raw data supporting the conclusions of this article will be made available by the authors without undue reservation.
